# Synergistic Design of ZnCo-MnO@NPC Cathode and ZIF-8@Zn Anode for High-Performance Aqueous Zinc-Ion Batteries

**DOI:** 10.3390/molecules31091429

**Published:** 2026-04-26

**Authors:** Rui Zhang, Xinhuan Zhang, Jialiang Li, Wenting Li, Huan Pang

**Affiliations:** 1 School of Biological and Chemical Engineering, Yangzhou Polytechnic University, Yangzhou 225009, China; 101547@yzpc.edu.cn (R.Z.);; 2 Yangzhou Key Laboratory of Food Safety and Health Technology, Yangzhou Polytechnic University, Yangzhou 225009, China; 3 Yangzhou Engineering Research Center of Agricultural Products Intelligent Measurement and Control & Cleaner Production (YZM2022127), Yangzhou Polytechnic University, Yangzhou 225009, China; 4 School of Chemistry and Chemical Engineering, Yangzhou University, Yangzhou 225009, China

**Keywords:** ZnCo co-doped, MnO@NPC, ZIF-8@Zn interface protective layer, Zinc ion battery

## Abstract

Manganese-based cathodes offer high capacity, low cost, and safety for aqueous zinc-ion batteries (AZIBs), yet suffer from Mn dissolution, Jahn–Teller distortion, and sluggish Zn^2+^ kinetics. Herein, a Zn/Co co-doped MnO nanoporous carbon composite (denoted as ZnCo-MnO@NPC) derived from a bimetallic ZnCoMn metal–organic framework (ZnCoMn-MOF-74) is successfully synthesized and proposed as a high-performance cathode to address these challenges. The introduction of Zn^2+^ increases the initial specific capacity of MnO, while Co doping effectively suppresses the Jahn–Teller distortion and improves the integrity of the structure. Furthermore, the nanoporous carbon matrix facilitates electrolyte infiltration and accelerates ionic transport. To further suppress dendrite growth and enhance cycling stability, a zeolitic imidazolate framework (ZIF-8) protective layer is engineered on the zinc anode (denoted as ZIF-8@Zn), effectively mitigating dendrite formation. The ZnCo-MnO@NPC//ZIF-8@Zn full cell demonstrates superior electrochemical performance, delivering 281.3 mAh g^−1^ at 0.1 A g^−1^ and retaining 98.7% of this value after 3500 long-term cycles at 2.0 A g^−1^, a remarkable finding that underscores its potential for high-performance energy storage. Collectively, this work highlights that transition metal ion doping represents an effective way to design efficient high-performance MOF-derived cathodes of AZIBs.

## 1. Introduction

The contradiction between the surging global energy demand and the limited supply of fossil fuels has driven researchers to develop advanced energy storage and conversion devices [1,2]. In the realm of electrochemical storage, aqueous zinc-ion batteries (AZIBs) are distinguished as a highly competitive technology due to their notable safety, cost-effectiveness, and environmental friendliness. Zinc metal is widely used as the anode material in energy storage systems. This prevalence stems from its favorable intrinsic characteristics, which include a substantial theoretical specific capacity about 820 mAh g^−1^ and an appropriately low redox potential about −0.76 V relative to the standard hydrogen electrode (vs. SHE) [3,4,5]. However, the sluggish Zn^2+^ diffusion kinetics induced by polarization effects and cathodic irreversible lattice distortions, coupled with dendrite growth at the Zn anode, collectively impede the commercialization of AZIBs for large-scale energy storage [6]. Consequently, full AZIBs integrating high-performance cathodes and dendrite-free Zn anodes offer a viable pathway to address these challenges. Specifically, to achieve AZIBs with high capacity and energy density, it is highly necessary to develop cathode materials with good performance.

Manganese-based metal–organic frameworks (Mn-MOFs) have emerged as ideal cathode precursors owing to their abundant topological architectures, tunable pore sizes, and tailorable morphologies, which facilitate ionic transport and expose more active sites. To date, MOF-derived MnO_2_, Mn_2_O_3_, and Mn_3_O_4_ have been successfully employed as cathode materials in AZIBs, demonstrating exceptional Zn^2+^ storage performance [7,8,9]. Among these, previous studies have demonstrated that MnO exhibits pronounced electrochemical activity in its low-oxidation state upon activation. To address the issues outlined above and improve the zinc storage properties of manganese oxides, transition metal ion doping has been recognized as a pivotal modification strategy. It achieves this enhancement through regulating the electronic configuration, while concurrently boosting the electrical conductivity and structural stability of the material [10,11,12,13]. For example, Li et al. introduced Fe^3+^ and Co^2+^ doped δ-MnO_2_ (denoted as FMO and CMO) as cathodes for AZIBs via a straightforward precipitation method. Both Fe^3+^ and Co^2+^ ions are capable of substituting a portion of Mn atoms, thereby maintaining the integrity of the framework structure [14]. In a separate study, Li and co-workers successfully fabricated carbon nanosheet arrays doped with multivalent cobalt ions (Co^2+/3+/4+^), which subsequently delivered an impressive discharge capacity of 362 mAh g^−1^ at 0.1 A g^−1^ as a Co-Mn_3_O_4_ cathode and demonstrated exceptional durability, with 80% capacity retention after 11,000 cycles at a higher rate of 2 A g^−1^ [15].

Despite these advances, the inherent limitations of manganese-based cathodes often cannot be fully mitigated through single metal ion doping [16,17,18,19,20]. To address this issue, bimetallic co-doping has emerged as a good strategy due to its multiple advantages, including stabilization of the crystal structure through synergistic effects, suppression of manganese dissolution, and introduction of oxygen vacancies, all of which contribute to superior electrochemical performance. Bimetallic doping brings more enhanced active sites, higher charge storage capacity and improved interfacial charge redistribution dynamics across heteroionic species to manganese-based cathode materials, thereby synergistically mitigating issues of structural instability and low specific capacity [21,22]. For example, Ma and co-workers synthesized Mo,P co-doped MnO_2_ nanoflowers as cathodes and demonstrated that the synergistic interaction between Mo and P dopants significantly preserves the structural integrity and electrochemistry kinetics of the Mo,P-MnO_2_ electrode during cycling, thereby suppressing capacity degradation and improving rate performance [23]. Nevertheless, reports on Mn-based Zn/Co co-doped cathodes derived from MOFs remain scarce in the literature, despite their potential for synergistic modulation of electrochemical performance through dual-cationic interactions [24,25,26].

In this study, a novel Zn/Co co-doped MnO nanoporous carbon composite (denoted as ZnCo-MnO@NPC) was synthesized via a one-pot calcination route using trimetallic ZnCoMn-MOF-74 as the sacrificial precursor. Researchers have conducted systematic investigations into how the incorporation of metal ions influences both the crystalline structure and electrochemical behavior of MnO. It is revealed that Co^2+^ doping alone markedly enhances the initial capacity and cycling stability of MnO@NPC. Moreover, a further improved synergistic effect is achieved when Zn^2+^ is additionally incorporated. The resulting Zn/Co co-doped MnO@NPC possesses a richer concentration of oxygen vacancies compared to its singly doped counterpart, which accelerates the reaction kinetics and underpins the enhanced electrochemical performance of the Mn-based oxide. Experimental results reveal that the synergistic interaction arising from Zn/Co co-doping contributes to both an increase in the initial capacity of the electrode material and an improvement in its cycling stability, owing to the effective suppression of Jahn–Teller distortion. Notably, the optimized ZnCo-MnO@NPC-800 cathode exhibits superior Zn^2+^ storage performance, exhibiting an enhanced specific capacity of 356.7 mAh g^−1^ upon testing at 0.1 A g^−1^ and retaining 84.8% at 2 A g^−1^ after 3000 cycles. Furthermore, a ZIF-8 protective layer was deposited onto the Zn anode surface to suppress dendrite growth and corrosion, enabling the assembly of full AZIBs with extended cycle life. When assembled into flexible pouch cells, the ZnCo-MnO@NPC-800 cathode, paired with a ZIF-8@Zn anode, delivers an impressive reversible charge–discharge capacity approaching 201.8 mAh g^−1^ at 0.1 A g^−1^.

## 2. Results and Discussion

### 2.1. Materials Characterization

**Figure 1:**
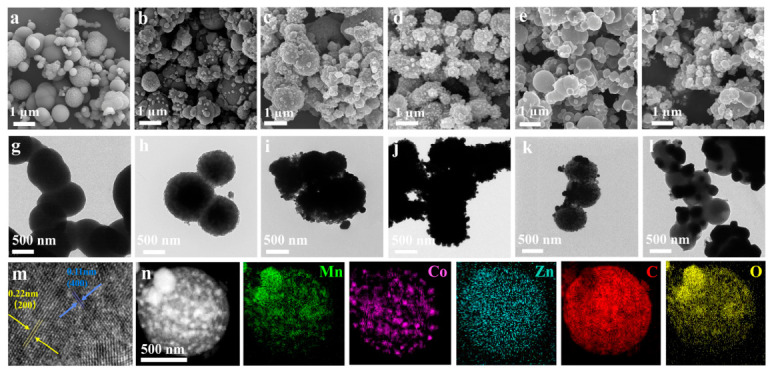
(**a**–**f**) SEM images and (**g**–**l**) TEM images of ZnCoMn-MOF-74, MnO@NPC, Co-MnO@NPC, ZnCo-MnO@NPC-600, ZnCo-MnO@NPC-700, ZnCo-MnO@NPC-800. (**m**) HRTEM images of ZnCo-MnO@NPC-800. (**n**) Elemental mappings of ZnCo-MnO@NPC-800.

Insights into the microstructural characteristics of ZnCoMn-MOF-74, ZnCo-MnO@NPC, Co-MnO@NPC, and MnO/C were gained through scanning electron microscopy (SEM) and transmission electron microscopy (TEM) methods. Figure 1a exhibits an irregular microsphere morphology of ZnCoMn-MOF-74, with an average diameter about 600 nm. The ZnCoMn-BTC-derived MnO@NPC presents a more uniform and smoother microspherical morphology, which is accompanied by an average particle size of roughly 500 nm (Figure 1b). This agglomeration behavior stems largely from the inherent structural characteristics of the ZnCoMn-MOF-74 precursor. Figure 1c shows the morphological features of Co-MnO@NPC, which exhibits a diameter of approximately 500 nm. Figure 1d–f shows the morphology of ZnCo-MnO@NPC-600, ZnCo-MnO@NPC-700, and ZnCo-MnO@NPC-800 with larger microspheres, with diameters of up approximately to 1 μm. Consequently, the incorporation of Zn/Co ions is instrumental in influencing the agglomeration behavior of Mn-MOF-74, thereby determining the surface morphology of the resulting microspheres following calcination.

High-resolution TEM (HRTEM) analysis was conducted to provide a more detailed examination of the surface morphology of ZnCo-MnO@NPC-800. Figure 1m clearly reveals a well-defined interface between the uniformly distributed ZnCo-MnO nanoparticles and the carbon-based amorphous matrix. Additionally, two distinct lattice fringe spacings, measuring 0.222 nm and 0.111 nm, are clearly resolved, corresponding to the (200) and (400) crystallographic planes of cubic MnO, respectively. This confirms that Zn/Co dual-doped MnO retains fine crystallinity. Figure 1n presents the corresponding elemental mappings. ZnCo-MnO nanoparticles, approximately 20 nm in size, are uniformly distributed on the carbon support. Besides, uniform signals of Co and Zn are detected, confirming that Co/Zn has been co-doped successfully. These findings collectively demonstrate that employing multi-metal MOFs as sacrificial templates represents a viable strategy for the synthesis of metal oxides with ion doping. This approach not only retains the pristine carbon architecture of the MOF precursor but also promotes a more uniform dispersion of the dopant ions throughout the material [27].

**Figure 2:**
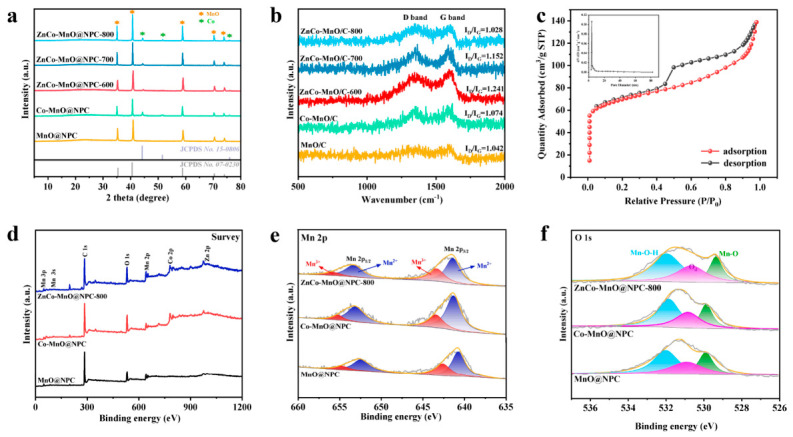
(**a**) XRD patterns and (**b**) Raman spectrum of MnO@NPC, Co-MnO@NPC, ZnCo-MnO@NPC-600, ZnCo-MnO@NPC-700, ZnCo-MnO@NPC-800. (**c**) Nitrogen adsorption/desorption isotherms and pore-size distribution curve of ZnCo-MnO@NPC-800. XPS spectra of the MnO@NPC, Co-MnO@NPC, ZnCo-MnO@NPC-800: (**d**) survey spectrum; (**e**) Mn 2p; (**f**) O 1s.

MOF-derived Mn-based oxides were studied by X-ray diffraction (XRD), with the resulting patterns shown in Figure 2a. In the pattern of pure MnO@NPC, five characteristic peaks can be identified at 34.9°, 40.6°, 58.7°, 70.2°, and 73.8°, which are characteristic of the cubic MnO phase and indicative of the (111), (200), (220), (222), and (311) planes, respectively. Which indicate a successful conversion from Mn-MOF-74 to MnO@NPC under an Ar atmosphere. In contrast to pure MnO@NPC, the Co-doped sample displays slightly reduced peak intensities along with increased full width at half maximum (FWHM), whereas the diffraction peak positions remain essentially unchanged. This indicates that the MnO phase is preserved after doping. However, the absence of obvious peak shifts suggests that XRD alone does not provide definitive evidence for substitutional doping, possibly due to the low dopant content and limited structural perturbation. Therefore, the incorporation of Zn/Co is discussed here in conjunction with elemental mapping and XPS results.

The Raman spectrum in Figure 2b shows an I_D_/I_G_ ratio of 1.028 for ZnCo-MnO@NPC-800, indicating a defect-rich carbon matrix with partial graphitic character rather than a highly graphitized structure. Compared with the undoped sample, the doped material may exhibit modified carbon ordering; however, the I_D_/I_G_ value still suggests the presence of substantial structural disorder. The specific surface area of ZnCo-MnO@NPC-800 was evaluated by Brunauer–Emmett–Teller (BET) analysis based on N_2_ adsorption–desorption isotherms, with the corresponding isotherm illustrated in Figure 2c. It can be concluded that the material possesses a BET surface area of approximately 209.7 m^2^ g^−1^. The BJH pore size distribution curve displays its average pore size is about 3.26 nm, confirming the presence of mesopores. The N_2_ adsorption/desorption isotherms, pore size distribution plots and pore porosity of MnO@NPC and ZnCo-MnO@NPC are provided in Figures S1–S2 and Table S1, respectively.

X-ray photoelectron spectroscopy (XPS) method was performed to characterize the chemical valence states present in the Co^2+^/Zn^2+^ co-doped MnO@NPC. Figure 2d reveals the existence of Mn, Zn, Co, O, and C in the survey spectrum. High-resolution Mn 2p spectra for the three samples are shown in Figure 2e. Deconvolution of the spectra reveals two spin–orbit doublets corresponding to Mn^2+^ (2p_3/2_ at 641.4 eV; 2p_1/2_ at 653.0 eV) and Mn^3+^ (2p_3/2_ at 643.1 eV; 2p_1/2_ at 654.9 eV), along with a satellite feature located at 647.0 eV. The fitted Mn^2+^/Mn^3+^ ratios were estimated to be 1.285, 1.498, and 1.417 for MnO@NPC, Co-MnO@NPC, and ZnCo-MnO@NPC-800, respectively. These results suggest that the introduction of Co and Zn influences the Mn valence-state distribution. In particular, Co doping appears to decrease the relative Mn^3+^ content, while Zn/Co co-doping leads to an intermediate Mn^2+^/Mn^3+^ ratio between those of MnO@NPC and Co-MnO@NPC. Such changes may be associated with the incorporation of Zn/Co species into or onto the MnO framework and may help alleviate Jahn–Teller distortion while retaining electrochemical activity [14,28]. However, the exact dopant concentration and substitution configuration cannot be conclusively determined from the present XPS results alone and require further quantitative compositional analysis.

As depicted in Figure 2f, deconvolution of the high-resolution O 1s spectrum yields three distinct peaks located at binding energy values of 530.0 eV, 531.3 eV, and 533.2 eV, corresponding to lattice-bound oxygen (Mn–O), oxygen vacancies (defective oxygen), and surface-adsorbed hydroxyl groups, respectively [14]. During calcination, carbon reacts with oxygen, extracting lattice oxygen and generating oxygen vacancies.

### 2.2. Application in Zinc Storage

**Figure 3:**
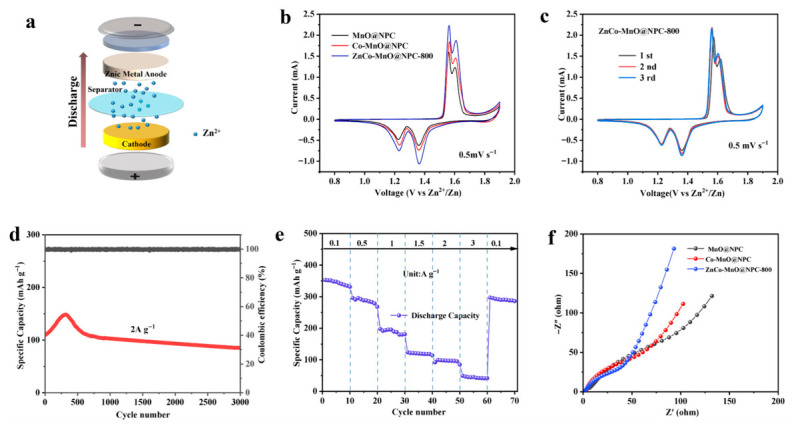
(**a**) Schematic illustration of aqueous Zn/ZnCo-MnO@NPC-800 battery, (**b**) CV curves of MnO@NPC, Co-MnO@NPC, and ZnCo-MnO@NPC electrodes at 0.5 mV s^−1^. (**c**) CV curves of ZnCo-MnO/@NPC-800. (**d**) Long-term cycling performance at 2.0 A g^−1^ and (**e**) rate performance of ZnCo-MnO@NPC-800. (**f**) EIS curves of MnO@NPC, Co-MnO@NPC and ZnCo-MnO@NPC-800.

For the purpose of evaluating the electrochemical behavior of the aqueous zinc-ion battery (AZIB), a coin-type cell was constructed consist of a ZnCo-MnO@NPC-800 cathode, a metallic zinc foil anode, and a mixed electrolyte including 2 M ZnSO_4_ and 0.2 M MnSO_4_. Figure 3a illustrates a schematic diagram of the AZIB configuration [29]. Figure 3b presents the cyclic voltammetry (CV) curves of ZnCo-MnO@NPC-800, Co-MnO@NPC, and MnO@NPC, recorded after three cycles at 0.5 mV s^−1^. All as-prepared samples exhibit two distinct pairs of redox characteristic peaks with reversible characteristics, which is commonly observed in manganese-based oxides. This similarity indicates that the incorporation of Zn/Co ions does not alter the fundamental crystal structure of MnO and preserves a comparable electrochemical reaction pathway. Compared to pristine MnO@NPC, both Co-MnO@NPC and ZnCo-MnO@NPC-800 demonstrate enhanced peak currents and integrated areas, confirming the positive effect of metal doping on electrochemical performance. Among them, ZnCo-MnO@NPC-800 exhibits the largest integrated area, further confirming its superior electrochemical activity. Furthermore, the prominent reduction peak of ZnCo-MnO@NPC-800 is a little shifted toward higher potential, narrowing the potential gap. This indicates that Zn^2+^/Co^2+^ co-doping alleviates MnO polarization and promotes favorable redox reaction progress.

To investigate the effect of Zn/Co co-doping on the activation of MnO, multi-cycle cyclic voltammetry measurements were conducted. The resulting CV curves for ZnCo-MnO@NPC-800, obtained over successive cycles at 0.5 mV s^−1^, are presented in Figure 3c. The first cycle reveals a characteristic oxidation peak at 1.58 V, which can be ascribed to an activation process involving Mn^2+^ dissolution and the creation of oxygen vacancies or defects. From the second cycle onward, the CV profiles stabilize, displaying two pairs of redox peaks. The two anodic peaks positioned at 1.54 V and 1.61 V result from the extraction of Zn^2+^ and H^+^ ions, respectively. Conversely, the cathodic peaks observed at 1.23 V and 1.37 V arise from the insertion of Zn^2+^ and H^+^ into the electrode material. These results confirm the good electrochemical reversibility of ZnCo-MnO@NPC-800. Along with the shift in peak position, a gradual increase in peak area was observed upon cycling, suggesting that MnO undergoes an activation process, evolving from an electrochemically inert state to an active one.

At 2.0 A g^−1^, the prolonged cycling performance of ZnCo-MnO@NPC-800 was assessed, as illustrated in Figure 3d. The electrode offers an initial specific capacity approaching 135.35 mAh g^−1^ and retains 84.8% of its original capacity after 3000 charge–discharge indicating stable long-term cycling performance [30]. The rate capability of the electrode was further assessed, as shown in Figure 3e. At stepwise increasing current densities of 0.1–3.0 A g^−1^, the ZnCo-MnO@NPC-800 electrode delivers specific capacities of 356.7, 222.9, 132.9, 101.9, 84.6, and 62.6 mAh g^−1^, respectively.

To further probe the electrochemical kinetics, electrochemical impedance spectroscopy (EIS) was conducted on freshly assembled coin cells, as presented in the Nyquist plots in Figure 3f. The charge transfer resistance (*R*ct) values for ZnCo-MnO@NPC-800, Co-MnO@NPC, and MnO@NPC were calculated to be 20.37, 27.53, and 51.80 Ω, respectively. Among the three manganese oxides, MnO@NPC exhibits a lower *R*ct owing to its conductive carbon framework. Furthermore, *R*ct is significantly changed upon doping of Co and Zn ions. Thus, Co/Zn co-doping results in a favorable modulation of the internal electronic configuration of MnO, which in turn accelerates the charge transfer process [26]. In addition to the beneficial effect of co-doping, the nanoporous carbon matrix of ZnCo-MnO@NPC-800 also plays a critical role in the observed electrochemical performance. The mesoporous structure provides efficient diffusion channels for Zn^2+^ ions, reducing ion transport resistance and contributing to the low charge transfer resistance (20.37 Ω) observed in EIS. Moreover, the relatively high surface area offers abundant active sites for Zn^2+^ adsorption, enhancing the specific capacity. The porous carbon framework also buffers volume changes during repeated Zn^2+^ intercalation/deintercalation, which helps maintain structural integrity over long-term cycling. These structure-performance relationships are consistent with previous reports on MOF-derived carbon materials for energy storage. The galvanostatic charge–discharge (GCD) profiles of MnO@NPC, Co-MnO@NPC, and ZnCo-MnO@NPC-800 at 0.1 A g^−1^ are shown in Figure S3. The pouch cell structure and its electrochemical performance are presented in Figure S4a–c, and a detailed comparison with previously reported cathode materials is summarized in Table S2.

**Figure 4:**
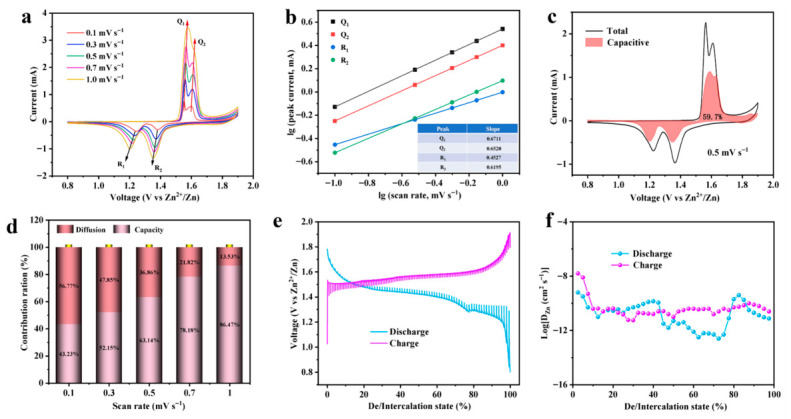
(**a**) CV curves of the ZnCo-MnO@NPC-800 electrode at various scan rates. (**b**) Linear fitting plots of log(*i*) versus log(*v*) for the selected redox peaks. (**c**) Capacitive contribution at 0.6 mV s^−1^. (**d**) Capacitive contribution ratios at different scan rates for the ZnCo-MnO@NPC-800 electrode. (**e**) GITT charge/discharge profiles of the ZnCo-MnO@NPC-800 electrode. (**f**) Corresponding Zn^2+^/H^+^ diffusion coefficients during the charge/discharge process derived from the GITT results.

To elucidate the capacitive vs. diffusion-controlled contributions to the overall charge storage of ZnCo-MnO@NPC-800, CV measurements were carried out at 0.1–1.0 mV s^−1^ scan rates, as depicted in Figure 4a [31]. Upon increasing the scan rate, both the peak area and peak intensity increase, while the overall CV profile remains well defined, illustrating the electrochemical stability of the ZnCo-MnO@NPC-800 material. As documented in earlier studies, the correlation between the peak current (i) and the scan rate (ν) satisfies a power-law equation, expressed as i = $\alpha \times \nu^{b}$ [32]. Four pairs of redox peaks are evident in Figure 4a. Based on the linear correlation between log(i) and log(ν) as fitted and shown in Figure 4b, the respective b-values of peaks Q_1_, Q_2_, R_1_, and R_2_ were calculated to be 0.6711, 0.531, 0.4527, and 0.6195, respectively. Since all b values are around 0.5, the electrochemical kinetics of the ZnCo-MnO@NPC-800 material are predominantly diffusion-controlled [33].

The kinetics of zinc ion storage at a fixed scan rate is controlled by both of capacitive and diffusion-controlled processes. This behavior can be quantitatively explained by *i* = k_1_ν + k_2_ν^1/2^, in which k_1_ν and k_2_ν^1/2^ denote the current contributions arising from surface capacitive effects and semi-infinite diffusion processes, respectively. The respective fractions of capacitive/diffusion-controlled progress for ZnCo-MnO@NPC-800 are presented in Figure 4d. At 0.1–1.0 mV s^−1^ scan rates, the capacitive-controlled fraction gradually rises from 43.23% to 86.41%. This trend indicates that capacitive control becomes increasingly dominant at higher scan rates, enabling the ZnCo-MnO@NPC-800 cathode to sustain a superior charge storage capacity under high current densities. At 0.5 mV s^−1^, the capacitive-controlled fraction reaches 59.70% (Figure 4d). Moreover, the fitted curve closely matches the total CV profile across the entire potential window.

To gain deeper insight into the intrinsic electrochemical kinetics and the co-embedding progress of H^+^ and Zn^2+^, the galvanostatic intermittent titration technique (GITT) method was employed to evaluate the ion diffusion coefficients, with the corresponding results illustrated in Figure 4e. As illustrated in Figure 4f, the coefficients of ZnCo-MnO@NPC-800 during the charge–discharge process fall from 10^−7^ to 10^−13^ cm^2^ s^−1^. The discharge curve features two distinct voltage plateaus located at approximately 1.45 V and 1.35 V, corresponding to plateau I and plateau II. Consistent with the CV analysis, plateau I can be attributed to the intercalation of H^+^ ions, whereas plateau II originates from the insertion of Zn^2+^ ions. Remarkably, the coefficient associated with plateau I is significantly larger than that of plateau II, suggesting more rapid kinetics for H^+^ insertion. This is further supported by the significantly lower overpotential of plateau I (48 mV) compared to plateau II (114 mV). These findings suggest that Zn/Co co-doping weakens the interaction between the host material and Zn^2+^/H^+^, while also suppressing the irreversible deintercalation from thermodynamically stable discharge products such as ZnMn_2_O_4_ and MnOOH, thereby facilitating ion diffusion.

Two well-defined discharge plateaus are observed in Figure 4e at approximately 1.28 V and 1.39 V, corresponding to the intercalation of Zn^2+^ ions accompanied by a variation in the oxidation state of manganese [34]. Furthermore, two weak charge plateaus are detected at 1.51 V and 1.59 V, owing to the deintercalation of Zn^2+^. To further elucidate the underlying electrochemical kinetics of the optimized ZnCo-MnO@NPC-800 sample, CV measurements were conducted at 0.1 to 1.0 mV s^−1^ over the potential range of 0.8–1.8 V (vs. Zn^2+^/Zn). The associated diffusion kinetics were then examined in detail.

**Figure 5:**
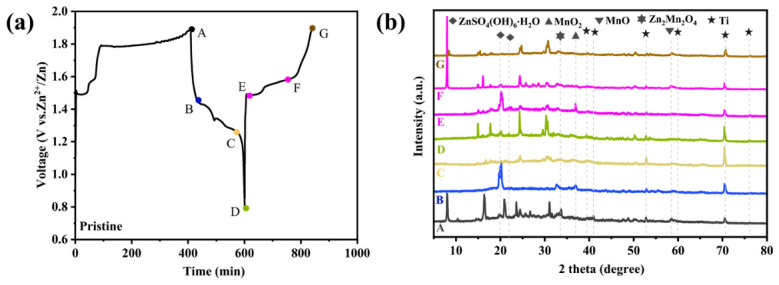
Research on the energy storage mechanism of the ZnCo-MnO@NPC-800 cathode. (**a**) A typical charge–discharge curve diagram, (**b**) Ex situ XRD patterns of the ZnCo-MnO@NPC-800 cathode under different states.

To elucidate the energy storage progress of MnO and clarify the part played by Zn/Co ions during electrochemical cycling, ex situ X-ray diffraction (XRD) patterns were studied. As shown in Figure 5a, an extended plateau at approximately 1.85 V is observed during the first charge cycle of ZnCo-MnO@NPC-800. However, the position of this plateau differs from those observed in subsequent cycles, suggesting an irreversible change in structure and oxidation state during the initial charge, likely associated with the activation reaction process of ZnCo-MnO@NPC-800 [35]. This process results in the formation of manganese vacancies, which in turn causes an elevation in the average oxidation state of manganese.

The five-pointed star marking presented in Figure 5b corresponds to the characteristic diffraction peaks of the stainless-steel current collector. When charged to point A (1.9 V, as shown in Figure 5a), ZnCo-MnO@NPC-800 retains the characteristic diffraction peaks of MnO, with no additional phases detected, indicating that the MnO crystal structure remains unchanged during the first charge cycle [36]. Upon discharging to 0.8 V (point C in Figure 5a), new diffraction peaks appear at 9.5°, 21.2°, and 32.9°, as revealed in Figure 5b. These peaks are indexed to the (002) plane of Zn_4_SO_4_(OH)_6_·6H_2_O (PDF#78-0247), the (110) plane of MnOOH (PDF#24-0713), and the (103) plane of ZnMn_2_O_4_ (PDF#24-1133), respectively. The formation of MnOOH and ZnMn_2_O_4_ serves as strong evidence for H^+^ and Zn^2+^ intercalation, respectively. Concurrently, the formation of Zn_4_SO_4_(OH)_6_·6H_2_O is ascribed to the localized elevation of OH^−^ concentration in the vicinity of the electrode, a consequence of H^+^ intercalation that ultimately induces its precipitation.

During subsequent charging, the diffraction intensities of these three phases gradually diminish. Upon charging to point E (1.9 V, as shown in Figure 5a,b), all characteristic peaks of the above phases disappear, confirming the reversibility of the H^+^/Zn^2+^ intercalation/deintercalation process and the structural stability of the MnO host. Accordingly, the electrochemical reactions of ZnCo-MnO@NPC-800 can be represented as follows:

ZnCo-MnO_2_ + H^+^ + e^−^ ⇌ ZnCo-MnOOH (1)

ZnCo-MnO_2_ + Zn^2+^ + 2e^−^ ⇌ ZnCo-ZnMn_2_O_4_ (2)

3Zn^2+^ + 6OH^−^ + ZnSO_4_ + 6H_2_O ⇌ Zn_4_SO_4_(OH)_6_·6H_2_O (3)

**Figure 6:**
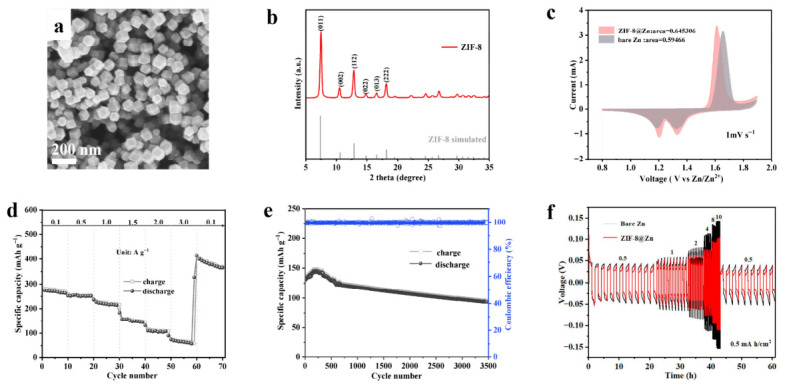
(**a**) SEM image of ZIF-8, zinc anode coating material; (**b**) XRD pattern of ZIF-8; (**c**) CV curve of ZnCo-MnO/C cathode with bare Zn and ZIF-8@Zn anode assembled full battery at 1 mV s^−1^; (**d**) full battery rate performance of ZIF-8@Zn modified anode; (**e**) full battery long cycle performance of ZIF-8@Zn anode at 2.0 A g^−1^; (**f**) rate performance of symmetric cells based on ZIF-8@Zn and Zn foil at a current density from 0.5 mA cm^−2^ to 10 mA cm^−2^ with the capacity limited to 0.5 mAh cm^−2^.

To suppress dendrite formation induced by the tip effect, ZIF-8 coating was applied to zinc foil via a suspension coating method; the resulting modified zinc foil is denoted as ZIF-8@Zn [37]. Figure 6a shows an SEM image of ZIF-8, and Figure 6b displays its corresponding morphology. To illustrate the mechanism of the ZIF-8 interfacial protective layer on electrochemical performance, a full battery was assembled while using ZIF-8@Zn as the anode and ZnCo-MnO@NPC-800 as the cathode. As can be seen from Figure 6c, the presence of similar redox peaks corresponding to H^+^/Zn^2+^ (de)intercalation reactions indicates that the ZIF-8 coating on the zinc anode hardly affects ion transport. Moreover, the integrated CV area for the cell employing the ZIF-8@Zn anode is calculated to be 0.6453, which exceeds that of the cell with bare Zn (0.5947). This enhancement can be ascribed to the ZIF-8 coating on the Zn anode, which promotes the capacitive contribution of the battery.

Figure 6d presents the rate performance of the ZnCo-MnO@NPC-800//ZIF-8@Zn full battery. Over a series of current densities, specifically 0.1, 0.5, 1.0, 1.5, 2.0, and 3.0 A g^−1^, the corresponding specific capacities are recorded as 281.3, 269.0, 177.8, 127.9, 105.3, and 81.1 mAh g^−1^. Compared to the above ZnCo-MnO@NPC-800 (shown in Figure 3e), the ZnCo-MnO@NPC-800//ZIF-8@Zn cell exhibits significantly increased rate performance, with a 72.2 mAh g^−1^ enhancement at 0.1 A g^−1^, further confirming the capacitive contribution of the interfacial ZIF-8 coating. This improvement is likely associated with the fact that the ZIF-8 protective layer leads to a reduction in polarization and charge transfer resistance.

To assess the effect of the ZIF-8 interfacial layer on the electrochemical performance of the full cell, long-term cycling tests were performed at 2.0 A g^−1^, as shown in Figure 6e. The ZnCo-MnO@NPC-800//ZIF-8@Zn full cell retains 98.7% of its initial capacity after 3500 cycles, indicating stable long-term cycling behavior. In addition, the improved interfacial stability of the Zn anode after ZIF-8 modification is further supported by the symmetric-cell measurements in Figure 6f, which exhibit lower polarization than the bare Zn symmetric cell. These results suggest that the ZIF-8 coating helps regulate Zn deposition and enhances anode interfacial stability.

To further evaluate the impact of the ZIF-8 interfacial protective layer on electrochemical stability, a symmetric cell was constructed using ZIF-8@Zn electrodes. As illustrated in Figure 6f, the ZIF-8@Zn symmetric cell exhibits substantially reduced voltage polarization relative to the bare Zn symmetric cell. At stepwise increasing current densities of 0.5–10.0 mA cm^−2^ and a fixed areal capacity of 0.5 mAh cm^−2^, the cell displays increasing overpotentials recorded as 34, 42, 53, 81, 104, and 112 mV, respectively. Strikingly, while the current density is switched back to 0.5 mA cm^−2^, subsequently the overpotential falls back to 27 mV—below its starting value—implying that an activation process takes place within the ZIF-8@Zn electrode during cycling. This activation phenomenon can be attributed to the repeated plating/stripping cycles, which enhance the porosity of the zinc anode and improve its interfacial wettability. These structural and surface modifications subsequently promote ion diffusion progress and enlarge the electrode-electrolyte contact area [38].

## 3. Conclusions

In summary, a Zn/Co co-doped MnO electrode material was successfully synthesized using a polymetallic MOF as a sacrificial precursor and subsequently applied in full aqueous zinc-ion batteries (AZIBs). The experimental results reveal that Zn doping mainly boosts the initial electrochemical reactivity of MnO, whereas Co doping (Co^2+^/Co^3+^) introduces multivalent states that actively participate in redox reactions throughout the cycling process. On one hand, this synergistic interplay between Zn and Co both boosts the specific capacity and effectively mitigates the Jahn–Teller distortion in reduction products, ensuring superior cycling stability. On the other hand, the co-doping strategy significantly improves both the ion diffusion kinetics and electronic conductivity of MnO, further contributing to its enhanced electrochemical performance. In addition, a ZIF-8 interfacial protective layer was introduced onto the Zn foil surface, which dramatically lowers the nucleation-deposition overpotential of zinc and suppresses the growth of dendrites. At 100 mA g^−1^, the as-assembled ZnCo-MnO@NPC//ZIF-8@Zn full cell offers a specific capacity as high as 281.3 mAh g^−1^. Remarkably, after 3500 cycles at 2.0 A g^−1^, a high-capacity retention of 98.7% is achieved, highlighting its exceptional long-term cycling stability. In summary, the results obtained provide valuable insights into the development of a viable design strategy for full AZIBs incorporating manganese-based cathodes and surface-protected anodes.

## Data Availability

The data that support the findings of this study are available from the corresponding author upon reasonable request.

## Supplementary Materials

The following supporting information can be downloaded at: https://www.mdpi.com/article/10.3390/molecules31091429/s1, Experimental section. Figure S1. The nitrogen adsorption/desorption isotherm and pore size distribution plot of MnO@NPC. Figure S2. The nitrogen adsorption/desorption isotherm and pore size distribution plot of ZnCo-MnO@NPC. Figure S3. Galvanostatic charge-discharge (GCD) profiles of the MnO@NPC,Co-MnO@NPC-800 and Co-MnO@NPC-800 at 0.1 A g^−1^. Figure S4. (a) Schematic illustration of the pouch cell structure, (b) CV curve of the ZnCo-MnO@NPC-800//Zn pouch cell at 0.1 mV s^−1^, (c) Galvanostatic charge-discharge curves of the ZnCo-MnO@NPC-800//Zn pouch cell at 0.1 A g^−1^. Table S1. Pore porosity of MnO@NPC and ZnCo-MnO@NPC-800. Table S2. Comparison of electrochemical performance of ZnCo-MnO@NPC-800//ZIF-8@Zn with previously reported cathode materials for AZIBs. Refs. [26,29,30,39,40,41,42,43,44,45,46,47,48,49] are cited in the Supplementary Materials.
