# Supplementary material for: Synergistic Design of ZnCo-MnO@NPC Cathode and ZIF-8@Zn Anode for High-Performance Aqueous Zinc-Ion Batteries
Source: Molecules. 2026 Apr 26;31(9):1429. doi: 10.3390/molecules31091429 (PMC13164788; doi:10.3390/molecules31091429)
Supplement: Supplementary file 1 [file molecules-31-01429-s001.zip › molecules-4201949-supplementary.pdf]

Supporting Information

1. Experimental section

1.1 Materials

All the chemicals in this study, including $MnCl_2·4H_2O$, 2,5-dihydroxyterephthalic acid, DMF, were used as received without further purification. All aqueous solutions were freshly prepared with high purity water (18 MΩ cm^{-1}).

1.2 Materials synthesis

1.2.1 Preparation of Cathode Materials

1.2.1.1 Synthesis of Mn-MOF-74

$MnCl_2·4H_2O$ (0.82 g, 4.14 mmol) and 2,5-dihydroxyterephthalic acid (H_4DOBDC, 123 mg, 0.621 mmol) were dissolved in 40 mL of N,N-dimethylformamide (DMF) and 5 mL of ethanol. The resulting solution was stirred for 30 min. Subsequently, the mixture was transferred into a 100 mL stainless steel autoclave and heated in an oven at 120 °C for 24 h. After cooling to room temperature, the product was collected by centrifugation, washed with DMF and ethanol, and dried at 60 °C for 12 h to obtain the final product.

1.2.1.2 Synthesis of MnCo-MOF-74

MnCo-MOF-74 was prepared following the same procedure as for Mn-MOF-74, except that a specific amount of $CoCl_2·6H_2O$ was added to the initial solution.

1.2.1.3 Synthesis of ZnCoMn-MOF-74

ZnCoMn-MOF-74 was also synthesized using a method identical to that of Mn-MOF-74, with the addition of specific amounts of $CoCl_2·6H_2O$ and $ZnCl_2$ to the initial solution.

1.2.1.4 Preparation of MnO@NPC Porous Carbon Composite

A porcelain boat containing the precursor Mn-MOF-74 sample was placed in a tube furnace. Under an argon atmosphere, the temperature was increased from room temperature to 700 °C at a heating rate of 2 °C/min and maintained at 700 °C for 2 h. The resulting composite material was denoted as MnO@NPC.

1.2.1.5 Preparation of Co-MnO@NPC Porous Carbon Composite

A porcelain boat containing the precursor CoMn-MOF-74 sample was placed in a tube furnace. Under an argon atmosphere, the temperature was increased from room temperature to 700 °C at a heating rate of 2 °C/min and held at 700 °C for 4 h. The resulting composite material was denoted as Co-MnO@NPC.

1.2.1.6 Preparation of ZnCo-MnO@NPC Porous Carbon Composites

Porcelain boats containing the precursor ZnCoMn-MOF-74 sample were placed in a tube furnace. Under an argon atmosphere, the temperature was increased from room temperature to target temperatures (600, 700, and 800 °C) at a heating rate of 2 °C/min. The samples were held at these respective temperatures for 4 h. Based on the different calcination temperatures, the resulting composite materials were denoted as ZnCo-MnO@NPC-600, ZnCo-MnO@NPC-700, and ZnCo-MnO@NPC-800, respectively.

1.2.2 Preparation of Zinc Anode Coating Material (ZIF-8)

Zinc nitrate hexahydrate (1.5 g) was weighed and completely dissolved in 70 mL of methanol to obtain Solution A. Subsequently, 2-methylimidazole (3.3 g) was weighed and completely dissolved in 70 mL of methanol to obtain Solution B. Solution A was then slowly added to Solution B, and the mixture was stirred for 24 h to complete the reaction. Afterward, the resulting milky slurry was centrifuged at 4000 rpm for 5 min, washed three times with methanol, and then dried in a vacuum oven at 80 °C overnight. Finally, the white crystalline sample was ground into a powder, dried, and stored for future use.

1.3 Battery Assembly

1.3.1 Preparation of the Cathode

The dried cathode active material and conductive agent (Super P) were placed in an agate mortar at mass ratios of 70% and 20%, respectively, and ground uniformly. The homogeneously mixed powder was transferred to a 5 mL glass sample vial. Subsequently, liquid polyvinylidene fluoride (PVDF, 5 wt.%) was added dropwise to the vial at a mass ratio of 10%, followed by the addition of an appropriate amount of N-methyl-2-pyrrolidone (NMP). The mixture was stirred for approximately 6-8 h until

a homogeneous and viscous slurry was obtained. The resulting slurry was uniformly coated onto titanium foil using a four-sided applicator and dried in a vacuum drying oven at 60 °C for 12 h.

1.3.2 Preparation of the Anode

The synthesized and dried ZIF-8 material and conductive agent (Super P) were placed in an agate mortar at mass ratios of 80% and 10%, respectively, and ground uniformly. The homogeneously mixed powder was transferred to a 5 mL glass sample vial. Then, liquid polytetrafluoroethylene (PTFE, 5 wt.%) was added dropwise to the vial at a mass ratio of 10%, along with an appropriate amount of N-methyl-2-pyrrolidone (NMP). The mixture was stirred for approximately 6-8 h until a homogeneous and viscous slurry was achieved. The resulting slurry was uniformly coated onto the surface of a zinc foil (which had been simply polished with sandpaper) using a four-sided applicator and dried in a vacuum drying oven for 12 h.

1.3.3 Assembly of Coin Cells

The titanium foil coated with the cathode material was cut into discs with a diameter of 12 mm using a disc cutter to serve as the cathode electrodes. The zinc foil, cleaned by ultrasonication in ethanol, was also cut into discs of the same diameter (12 mm) to serve as the anode electrodes. Glass fiber membranes were used as separators, and a solution of 2 M $ZnSO_4$ + 0.2 M $MnSO_4$ was used as the electrolyte for the CR2023-type coin cells. The cells were assembled in an air atmosphere in the following order: positive case, cathode electrode, electrolyte, separator, zinc anode, spacer, spring, and negative case.

The assembly procedure for the full cells was identical to that of the coin cells described above, using the same electrolyte. However, the anode was replaced with a zinc foil coated with the ZIF-8 material (ZIF-8@Zn).

1.3.4 Assembly of Pouch Cells

The dried cathode sheet was cut into a shape as illustrated in the figure below. The elongated strip portion served as the tab for the pouch cell. Excess active material was wiped off from the edges, leaving the coated area in a regular rectangular or square shape (record the length as *a* cm and the width as *b* cm). Subsequently, a zinc foil

was cut into the same shape as the cathode to serve as the anode. A solution of 2 M $ZnSO_4$ + 0.2 M $MnSO_4$ was used as the electrolyte. The cathode, a specialized glass fiber separator for pouch cells, and the zinc anode were stacked in sequence and transferred into an aluminum-plastic film. An electrolyte volume of ab/1.1304 × 100 μL was added between the cathode and the zinc anode. The pouch cell was then sealed using a sealing machine. The assembled pouch cells were allowed to rest for 12 h before proceeding with further electrochemical performance tests.

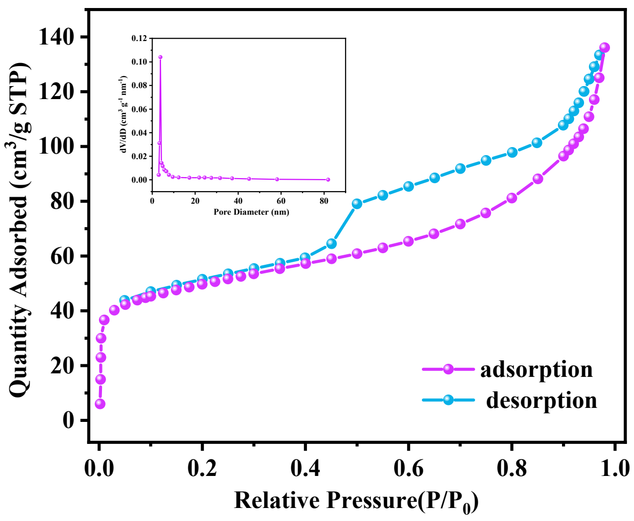

Figure S1. The nitrogen adsorption/desorption isotherm and pore size distribution plot of MnO@NPC;

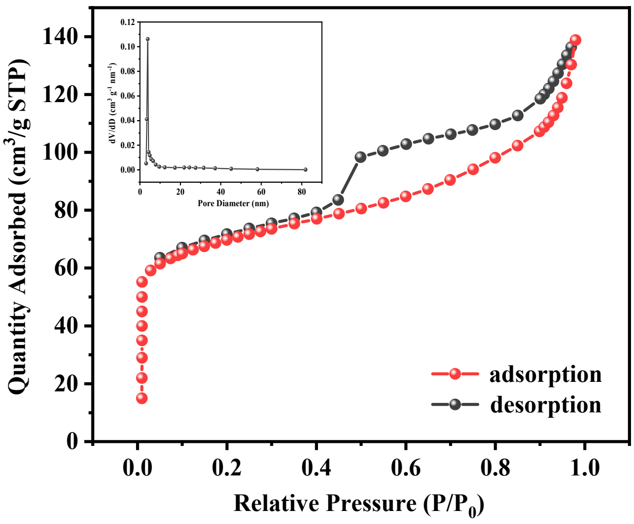

Figure S2. The nitrogen adsorption/desorption isotherm and pore size distribution plot of ZnCo-MnO@NPC.

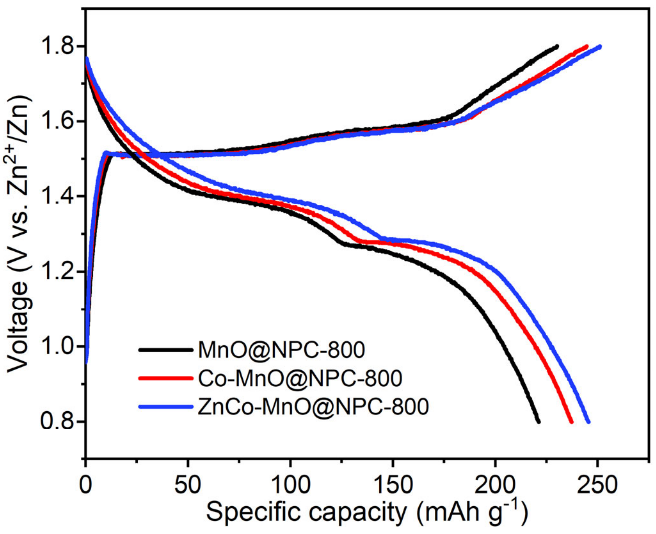

Figure S3. Galvanostatic charge-discharge (GCD) profiles of the MnO@NPC,Co-MnO@NPC-800 and Co-MnO@NPC-800 at 0.1 A g^{-1}.

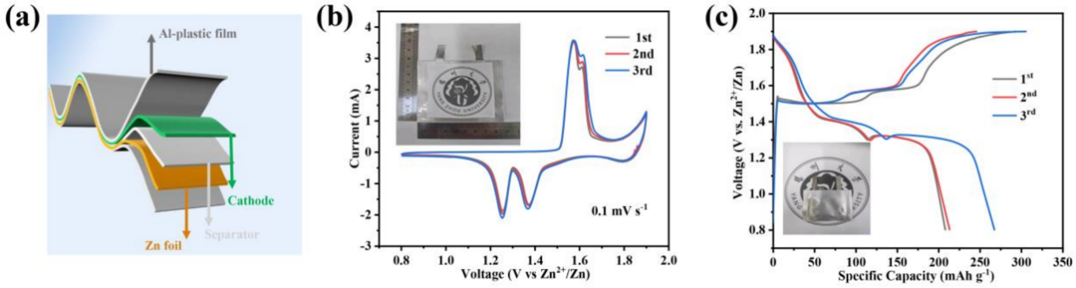

Figure S4. (a) Schematic illustration of the pouch cell structure, (b) CV curve of the ZnCo-MnO@NPC-800//Zn pouch cell at 0.1 mV s^{-1}, (c) Galvanostatic charge-discharge curves of the ZnCo-MnO@NPC-800//Zn pouch cell at 0.1 A g^{-1}.

Table S1. Pore porosity of MnO@NPC and ZnCo-MnO@NPC-800

Sample	S_{BET} (m^2/g)	Pore volume (cm^3/g)	average pore size (nm)
MnO@NPC	155.405	0.17	3.827
ZnCo-MnO@NPC-800	209.675	0.19	3.259

Table S2. Comparison of electrochemical performance of ZnCo-MnO@NPC-800//ZIF-8@Zn with previously reported cathode materials for AZIBs.

Samples	Specific capacity	Rate capability	Cycling stability	Ref
MnO/C@rGO	/	318.7 mAh g^{-1} at 0.2 A g^{-1}	0.5 A g^{-1}, 300 cycles, 170.6 mAh g^{-1}	1
MnO@CNFs	430 mAh g^{-1} at 0.1 A g^{-1}	420.1 mAh g^{-1} at 0.1 A g^{-1}	1.0 A g^{-1}, 2500 cycles, ~120 mAh g^{-1}	2
2D MnO/C	120.2 mAh g^{-1} at 0.5 A g^{-1}	230 mAh g^{-1} at 0.1 A g^{-1}	0.5 A g^{-1}, 900 cycles, 155.1 mAh g^{-1}	3
MnO-CNT@C_3N_4	209 mAh g^{-1} at 0.8 A g^{-1}	101 mAh g^{-1} at a 3 A g^{-1}	3 A g^{-1}, 500 cycles, ~100 mAhg^{-1}	4
MnO/ZnO/C	362 mAh g^{-1} at 1.20 A g^{-1}	191 mAh g^{-1} at 1.20 A g^{-1}	0.06 A g^{-1}, 500 cycles, ~350 mAh g^{-1}	5
Spheroidal MnO@C	412.4 mAh g^{-1} at 0.1 A g^{-1}	165.9 mAh g^{-1} at 3.0 A g^{-1}	3.0 A g^{-1}, 1000 cycles, 160 mAh g^{-1}	6
MnO_2/MnO@C	165 mAh g^{-1} at 0.5 A g^{-1}	110 and 100 mAh g^{-1} at 0.8 and 1 A g^{-1}	0.5 A g^{-1}, 200 cycles, 165 mAh g^{-1}	7
Commercial MnO particles	330 mAh g^{-1} at 0.1 A g^{-1}	267 mAh g^{-1} at 0.1 A g^{-1}	0.3 A g^{-1}, 300 cycles, ~275 mAh g^{-1}	8
MnO/C	336.8 mAh g^{-1} at 0.1 A g^{-1}	237 mAh g^{-1} at 0.1 A g^{-1}	1.0 A g^{-1}, 10000 cycles, 61.7 mAh g^{-1}	9
MnO@CC	192 mAh g^{-1} at 0.2 A g^{-1}	300 mAh g^{-1} at 100 mA g^{-1}	0.1 A g^{-1}, 300 cycles, 284 mAh g^{-1}	10
MnO hexagonal nanoplates	292 mAh g^{-1} at 0.1 A g^{-1}	260 mAh g^{-1} at 0.1 A g^{-1}	1.0 A g^{-1}, 1000 cycles, ~75 mAh g^{-1}	11
ZnCo-MnO/C	/	365 mAh g^{-1} at 0.1 A g^{-1}	3 A g^{-1}, 3000 cycles, ~80 mAh g^{-1}	12
MnO	288 mAh g^{-1} at 0.1 A g^{-1}	175 mAh g^{-1} at 0.1 A g^{-1}	0.5 A g^{-1}, 300 cycles, 112.3 mAh g^{-1}	13
MnO-Co@NC	192.3 mAh g^{-1} at 200 mA g^{-1}	200 mAh g^{-1} at 200 mA g^{-1}	2 A g^{-1}, 450 cycles, ~40 mAh g^{-1}	14
ZnCo-MnO@NPC-800	/	281.3 mAh g^{-1} at 0.1 A g^{-1}	2 A g^{-1}, 3500 cycles, ~88 mAh g^{-1}	This work

[1] H. Zhang, Y. Zhang, X. Li, J. Zhang, Y. He, X. Yang, J. Xu, D. Jia, J. Liu, In situ synthesis of MnO/C nanoparticles anchored on reduced graphene oxide as high-performance zinc ion battery cathode with enhanced zinc storage performance, Journal of Energy Storage 87 (2024) 111331.
[2] Y. He, Y. Pu, Y. Zheng, B. Zhu, P. Guo, X. Zhang, L. He, X. Wan, H. Tang, Carbon nanofiber-coated MnO composite as high-performance cathode material foraqueous zinc-ion batteries, Journal of Physics and Chemistry of Solids 184 (2024) 111669.
[3] Z.-X. Zhu, Z.-W. Lin, Z.-W. Sun, P.-X. Zhang, C.-P. Li, R. Dong, H.-W. Mi, Deciphering

H^+/Zn^{2+} co-intercalation mechanism of MOF-derived 2D MnO/C cathode for long cycle life aqueous zinc-ion batteries, Rare Metals 41(11) (2022) 3729-3739.
[4] X. Xiao, T. Wang, Y. Zhao, W. Gao, S. Wang, A design of MnO-CNT@C_3N_4 cathodes for high-performance aqueous zinc-ion batteries, Journal of Colloid and Interface Science 642 (2023) 340-350.
[5] E. Shangguan, L. Wang, Y. Wang, L. Li, M. Chen, J. Qi, C. Wu, M. Wang, Q. Li, S. Gao, Recycling of zinc− carbon batteries into MnO/ZnO/C to fabricate sustainable cathodes for rechargeable zinc-Ion batteries, ChemSusChem 15(15) (2022) e202200720.
[6] C. Yin, C. Pan, Y. Pan, J. Hu, Hierarchical spheroidal MOF-derived MnO@C as cathode components for high-performance aqueous zinc ion batteries, Journal of Colloid and Interface Science 642 (2023) 513-522.
[7] W. Jiang, H. Shi, X. Xu, J. Shen, Z. Xu, R. Hu, MnO stabilized in carbon-veiled multivariate manganese oxides as high-performance cathode material for aqueous Zn-ion batteries, Energy & Environmental Materials 4(4) (2021) 603-610.
[8] J. Wang, J.-G. Wang, H. Liu, Z. You, C. Wei, F. Kang, Electrochemical activation of commercial MnO microsized particles for high-performance aqueous zinc-ion batteries, Journal of Power Sources 438 (2019) 226951.
[9] K. Sun, J. Pang, Y. Zheng, F. Xing, R. Jiang, J. Min, J. Ye, L. Wang, Y. Luo, T. Gu, Oxygen vacancies enriched MOF-derived MnO/C hybrids for high-performance aqueous zinc ion battery, Journal of Alloys and Compounds 923 (2022) 166470.
[10] Q. Tan, Y. Song, X. Zhou, B. Yu, J. Song, Y. Liu, Electrochemically induced defects promotional high-performance binder-free MnO@CC cathodes for flexible quasi-solid-state zinc-ion battery, ACS Applied Energy Materials 5(12) (2022) 15510-15519.
[11] Z. You, H. Liu, J. Wang, L. Ren, J.-G. Wang, Activation of MnO hexagonal nanoplates via in situ electrochemical charging toward high-capacity and durable Zn-ion batteries, Applied Surface Science 514 (2020) 145949.
[12] K. Sun, Y. Shen, J. Min, J. Pang, Y. Zheng, T. Gu, G. Wang, L. Chen, MOF-derived Zn/Co co-doped MnO/C microspheres as cathode and Ti_3C_2@Zn as anode for aqueous zinc-ion full battery, Chemical Engineering Journal 454 (2023) 140394.
[13] W. Li, X. Gao, Z. Chen, R. Guo, G. Zou, H. Hou, W. Deng, X. Ji, J. Zhao, Electrochemically activated MnO cathodes for high performance aqueous zinc-ion battery, Chemical Engineering Journal 402 (2020) 125509.
[14] A. Samanta, B.K. Barman, S. Mallick, C.R. Raj, Three-dimensional nitrogen-doped graphitic carbon-encapsulated MnO-Co heterostructure: a bifunctional energy storage material for Zn-Ion and Zn–Air batteries, ACS Applied Energy Materials 3(10) (2020) 10108-10118.
